# Supplementary material for: OsMAPK6 phosphorylation and CLG1 ubiquitylation of GW6a non-additively enhance rice grain size through stabilization of the substrate
Source: Nat Commun. 2024 May 21;15:4300. doi: 10.1038/s41467-024-48786-0 (PMC11109111; doi:10.1038/s41467-024-48786-0)
Supplement: Supplementary file 1 — Supplementary Information [file 41467_2024_48786_MOESM1_ESM.pdf]

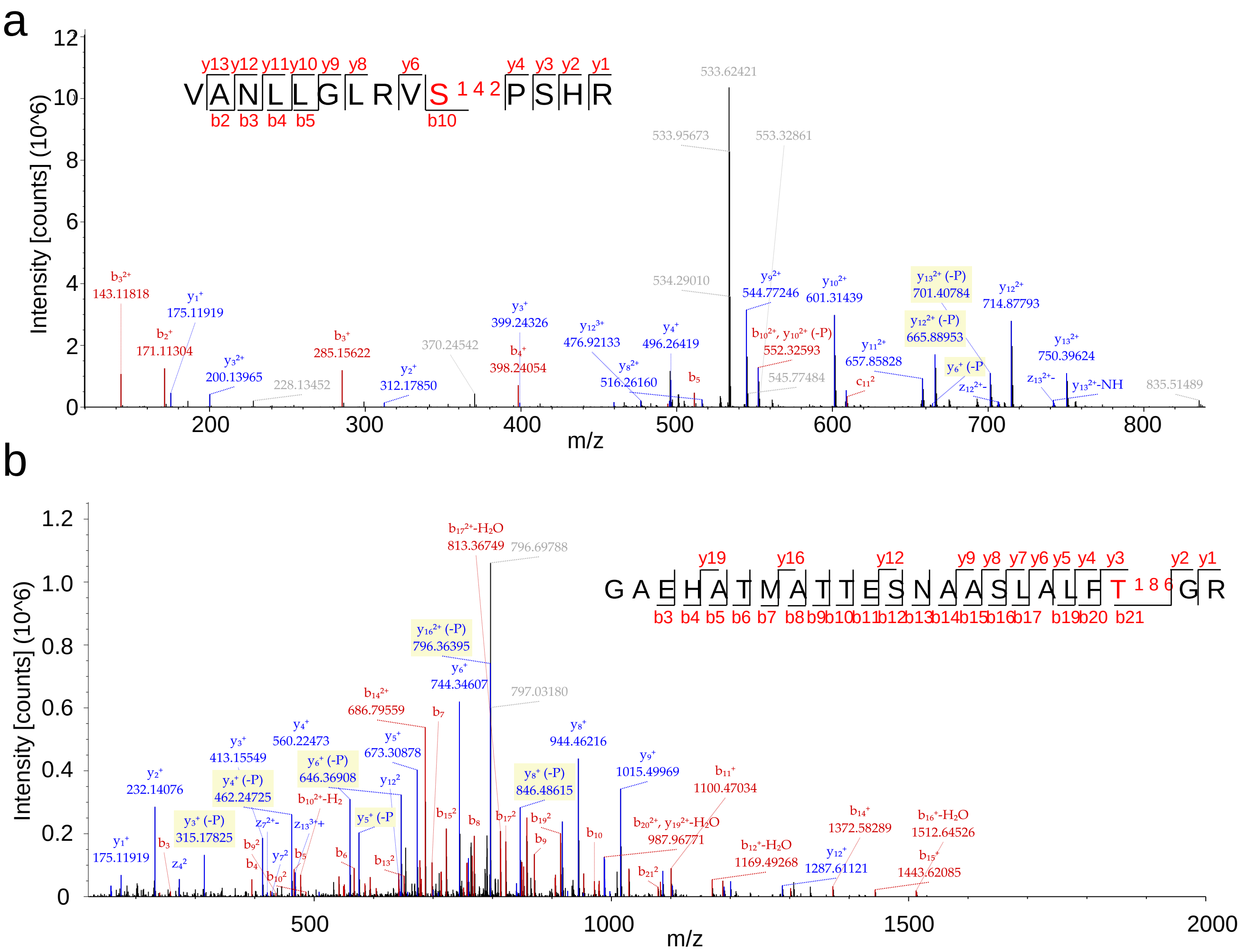

Supplementary Fig. 1 Identification of two GW6a phosphorylation sites targeted by OsMAPK6. MS/MS spectra for two phosphopeptides of GW6a showing in vitro OsMAPK6 phosphorylation sites of GW6a at S142 (**a**) and T186 (**b**).

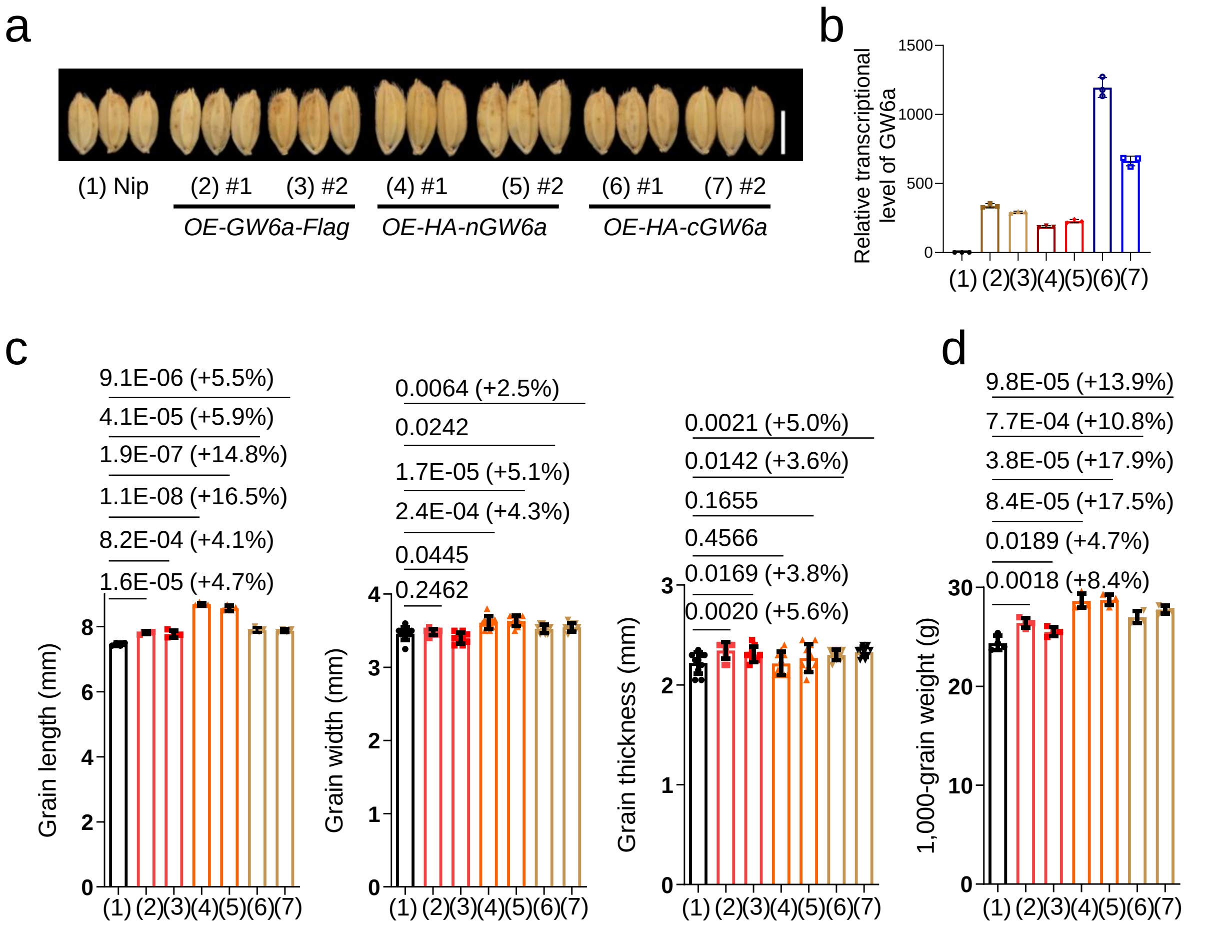

Supplementary Fig. 2 *OE-HA-nGW6a* has much longer and heavier grains than that of *OE-HA-cGW6a*. (**a**) Mature grains of Nipponbare (Nip, control), *OE-GW6a-Flag*, *OE-HA-nGW6a* (*p35S::HA-nGW6a*), and *OE-HA-cGW6a*. (**b**) Relative transcriptional expression of *GW6a* in Nip and the transgenic young panicles shown in (A). The *UBQ5* was used as the internal reference gene. Data are shown as mean ± SD. Three biological replicates are performed for each assay. (**c, d**) Comparisons of grain length (n = 4), grain width (n = 10), grain thickness (n = 10) (**c**), and 1,000-grain weight (n = 4) (**d**) between Nip and *OE-HA-nGW6a*, *OE-HA-cGW6a*. Data in (**c** and **d**) are mean ± SD, and P values were obtained by one-way Student's t-test compared with the corresponding controls.

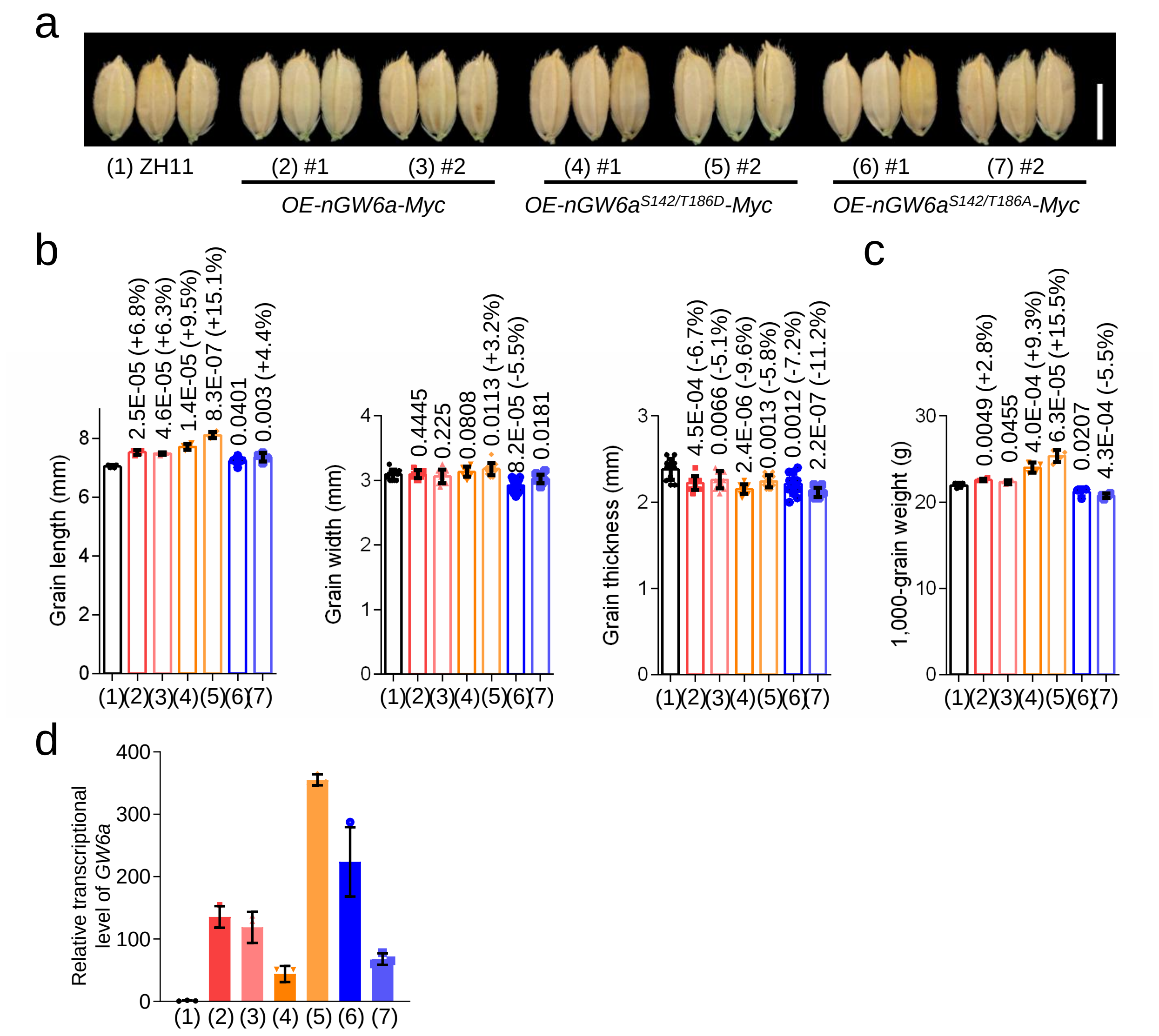

Supplementary Fig. 3 Phosphorylation of GW6a by OsMAPK6 presumably involves in the regulation of grain size (length). (**a**) Mature grains of ZH11, *OE-nGW6a-Myc*, *OE-nGW6a*$^{S142D/T186D}$*-Myc*, and *OE-nGW6a*$^{S142A/T186A}$*-Myc*. Scale bar = 5 mm. (**b, c**) Comparisons of grain length (n = 4), grain width (n = 12), grain thickness (n = 12) (**b**), and 1,000-grain weight (n = 4) (**c**) between Nip and *OE-nGW6a-Myc*, *OE-nGW6a*$^{S142/T186D}$*-Myc*, and *OE-nGW6a*$^{S142/T186A}$*-Myc*. Data are shown as mean ± SD. The one-way Student's t-test was used to generate the P values. (**d**) Relative transcriptional expression of GW6a in ZH11, *OE-nGW6a-Myc*, *OE-nGW6a*$^{S142/T186D}$*-Myc*, and *OE-nGW6a*$^{S142/T186A}$*-Myc* young panicles. Data are shown as mean ± SD. Three biological replicates are performed for each assay.

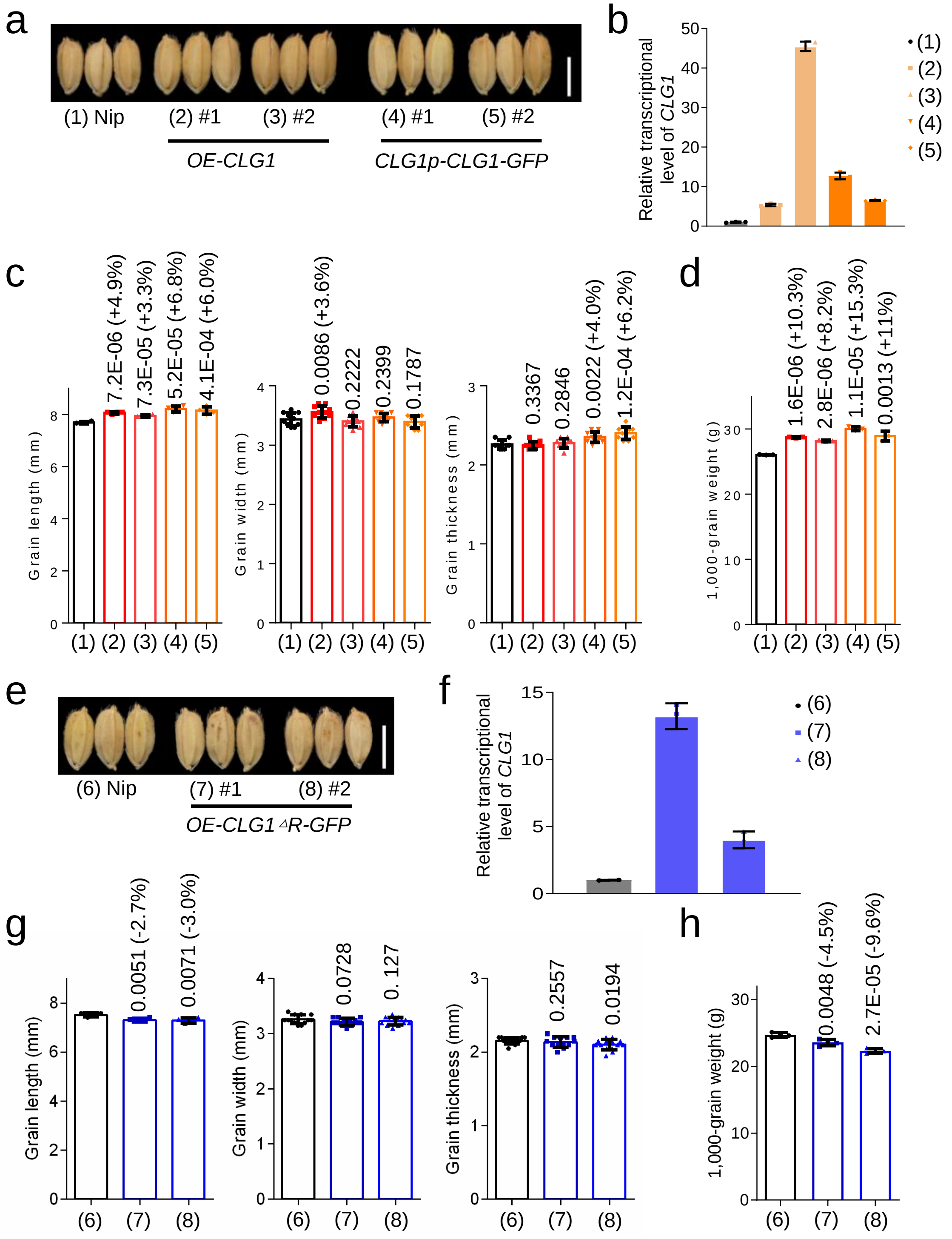

Supplementary Fig. 4 *CLG1* is a positive regulator of grain size (length). (**a**) Mature grains of Nip, *OE-CLG1s* and *CLG1p-CLG1-GFPs*. Scale bar = 5 mm. (**b**) Relative transcriptional expression of *CLG1* in Nip, *OE-CLG1s* and *CLG1p-CLG1-GFPs* shown in (**a**). (**c, d**) Comparisons of grain length (n = 4), grain width (n = 10), grain thickness (n = 10) (**c**), and 1,000-grain weight (n = 3) (**d**) between Nip and *OE-CLG1s* and *CLG1p-CLG1-GFPs*. (**e**) Mature grains of Nip and *OE-CLG1⊿R-GFPs*. Scale bar = 5 mm. (**f**) Relative transcriptional expression of *CLG1* in Nip and *OE-CLG1⊿R-GFPs* young panicles. Three replicates are performed for each assay in (**b** and **f**). (**g, h**) Comparisons of grain length (n = 4), grain width (n = 13), grain thickness (n = 13) (**g**), and 1,000-grain weight (n = 3) (**h**) between Nip and *OE-CLG1⊿R-GFPs*. Data are shown as mean ± SD (**b-d** and **f-h**), and the one-way Student's *t*-test was used to generate the *P* values in (**c**, **d**, **g**, and **h**).

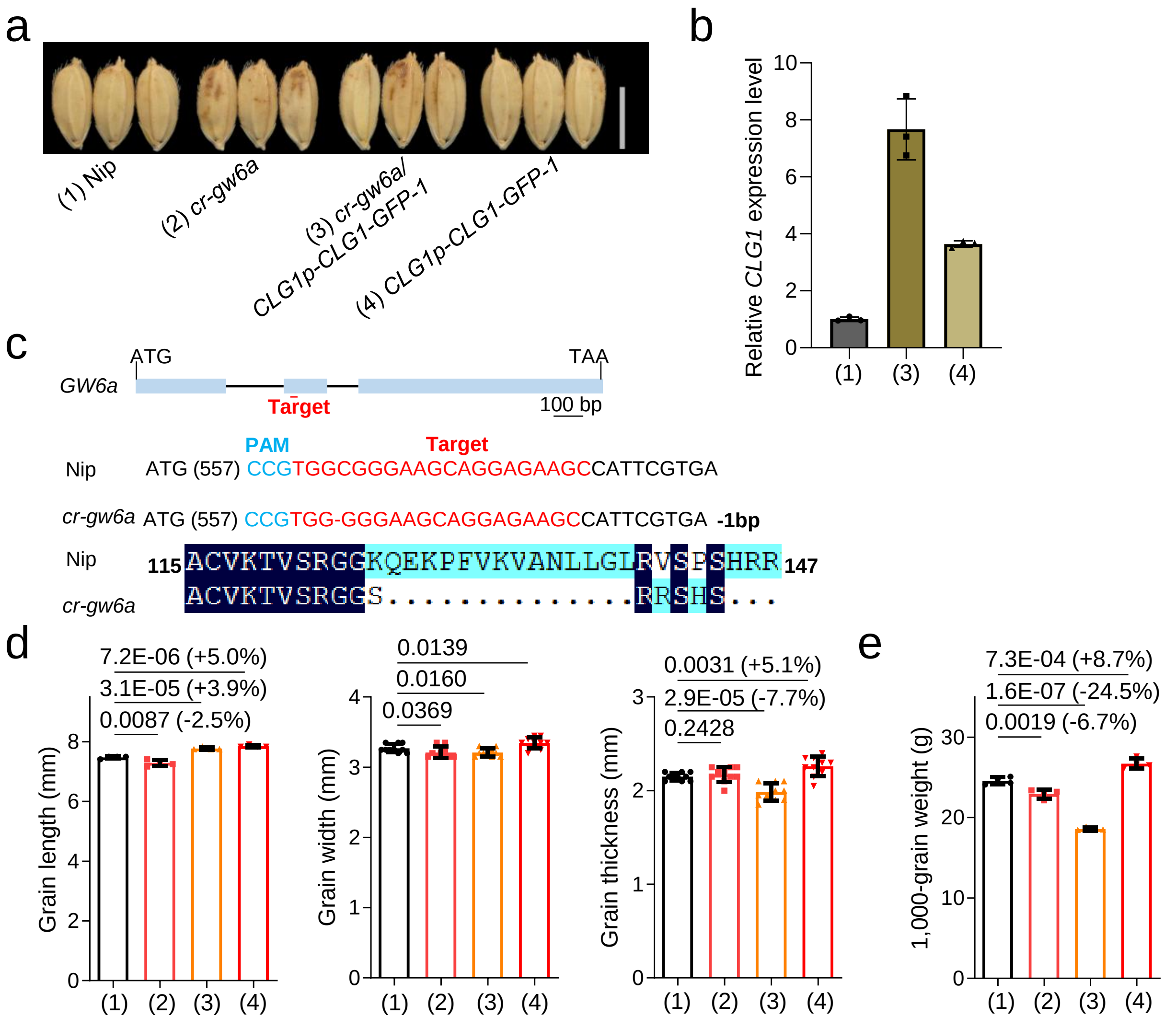

Supplementary Fig. 5 *CLG1* functions in a common genetic pathway with and upstream of *GW6a* to regulate grain length. (**a**) Mature grains of Nipponbare (Nip), *cr-gw6a*, *cr-gw6a/CLG1p-CLG1-GFP-1*, and *CLG1p-CLG1-GFP-1*. Scale bar = 5 mm. (**b**) Relative expression of *CLG1* in Nip and the transgenic young panicles shown in (**a**). The *UBQ5* was used as the internal reference gene (n = 3). (**c**) Molecular evidence for gene edition of GW6a by a CRISPR/Cas9 approach (the genotype of *cr-gw6a*). (**d, e**) Phenotypic comparisons of grain length (n = 4), grain width (n = 10), grain thickness (n = 10), and 1,000-grain weight (n = 4) in (**a**). Data are shown as mean ± SD (**b**, **d** and **e**), and the one-way Student's t-test was used to generate the P values in (**d** and **e**).

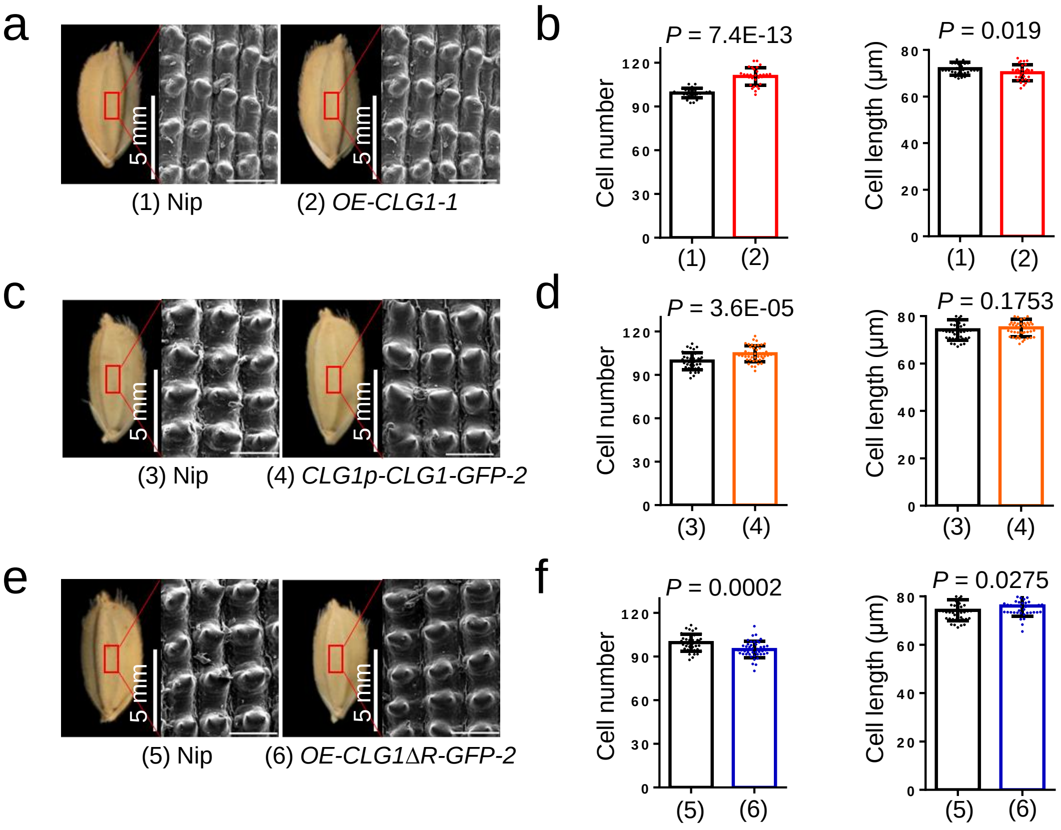

Supplementary Fig. 6 *CLG1* regulates grain size (length) through alteration of cell number in spikelet hulls. (**a**) Scanning electron microscopic analyses of the outer surfaces of Nip and *OE-CLG1-1* spikelet hulls. (**b**) Cell number (*n* = 30) and cell length (*n* = 30) of the outer epidermal cell as indicated in (**a**). (**c**) Scanning electron microscopic analyses of the outer surfaces of Nip and *CLG1p-CLG1-GFP-2* spikelet hulls. (**d**) Cell number (*n* = 44) and cell length (*n* = 44) of the outer epidermal cell as indicated in (**c**). (**e**) Scanning electron microscopic analyses of the outer surfaces of Nip and *OE-CLG1ΔR-GFP-2* spikelet hulls. Scale bars = 90 μm in (**a**, **c**, and **e**). (**f**) Cell number (*n* = 45) and cell length (*n* = 45) of the outer epidermal cell as indicated in (E). Data are shown as mean ± SD, and the one-way Student's *t*-test was used to generate the *P* values in (**b**, **d**, and **f**).

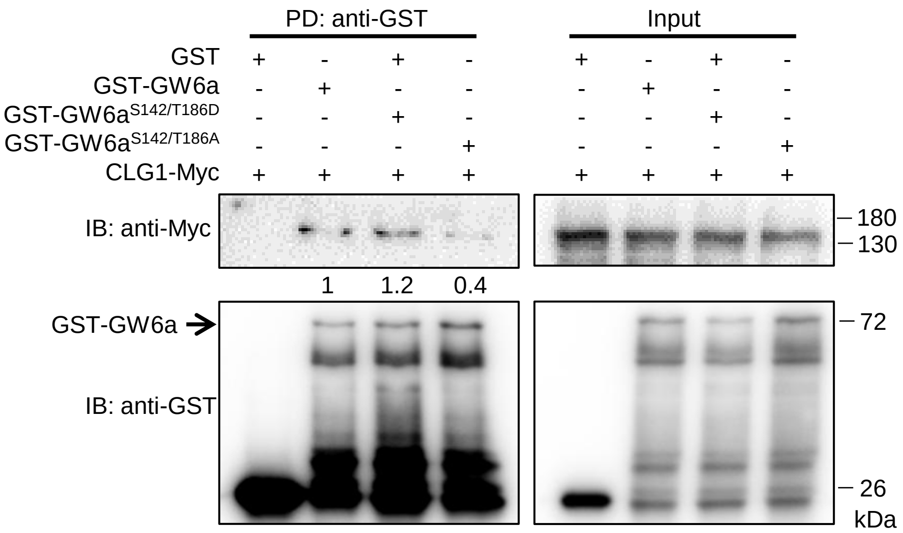

Supplementary Fig. 7 Phosphorylation of GW6a at S142/T186 enhances its interaction with CLG1 *in vitro*. Pull-down assays showing that the phosphorylation of GW6a by OsMAPK6 alters its interaction with CLG1. The experiment was repeated at least two times will similar results.

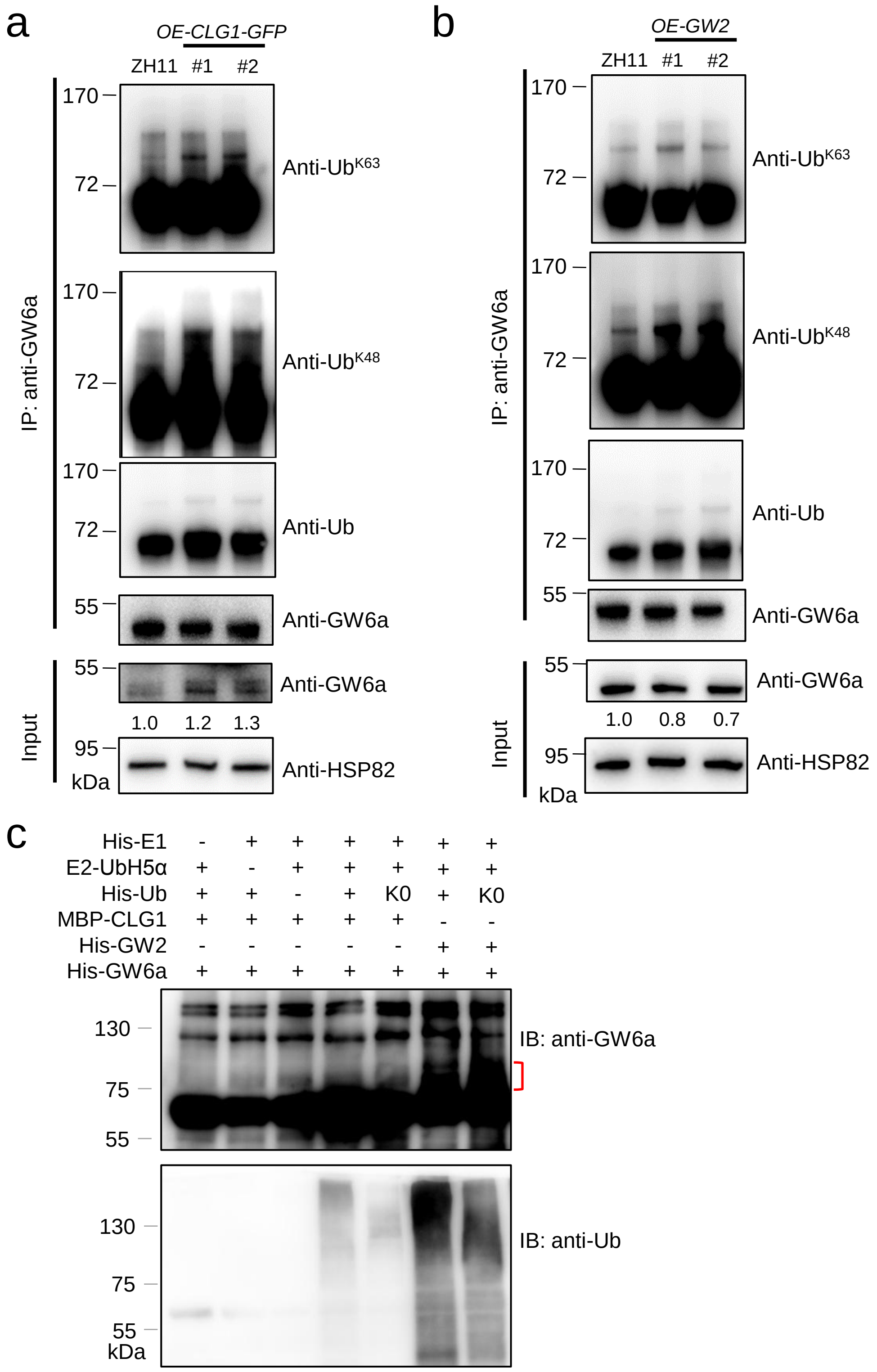

Supplementary Fig. 8 CLG1 and GW2 monoubiquitylate and polyubiquitylate GW6a, whereas the altered polyubiquitylation chain types differ. (**a**) Immunoblot assays showing that the *OE-CLG1-GFP* transgenic plants d isplay obviously enhanced levels of both of the K63 and K48-linked polyubiquitin chains relative to the c orresponding control. (**b**) Immunoblot assays showing that the *OE-GW2* transgenic plants have clearly enhanced le vels of the K48-linked polyubiquitin chains relative to the corresponding control. (**c**) CLG1 and GW2 p olyubiquitylate and mono-ubiquitylate GW6a *in vitro*. UbK0 was produced by altering the seven lysine residues of ub iquitin to arginine to prevent the forming of polyubiquitylation chains. The experiments in **a**-**c** were repeated a t least two times will similar results.

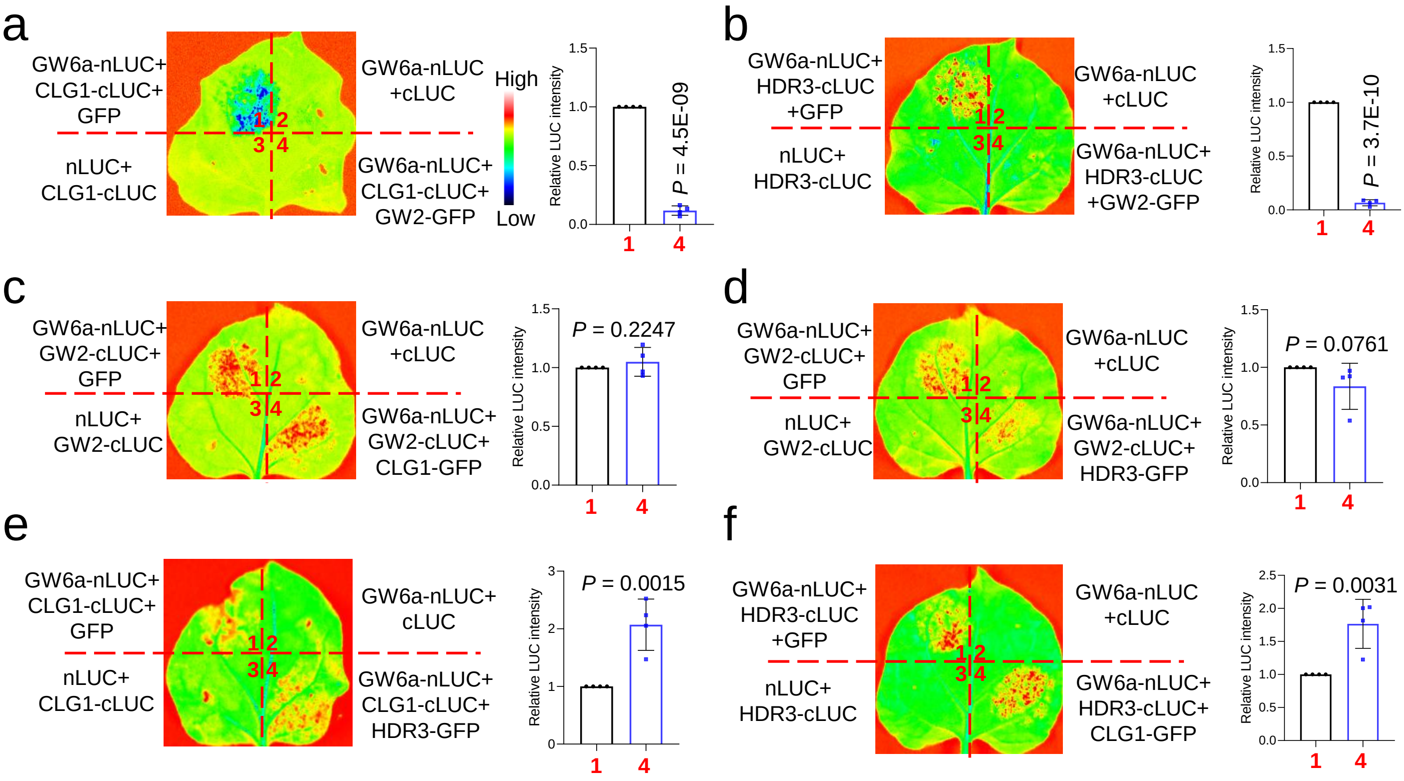

Supplementary Fig. 9 CLG1 and HDR3 cooperate with but compete with GW2 for the interaction with GW6a. Split firefly luciferase complementation assays of GW2, CLG1, HDR3, and GW6a in tobacco leaves, and quantification of LUC intensity using a Image J software. Bars represent means ± SD. The one-way Student's *t* test was used to generate the *P* values (*n* = 4).
