# Supplementary material for: OsMAPK6 phosphorylation and CLG1 ubiquitylation of GW6a non-additively enhance rice grain size through stabilization of the substrate
Source: Nat Commun. 2024 May 21;15:4300. doi: 10.1038/s41467-024-48786-0 (PMC11109111; doi:10.1038/s41467-024-48786-0)
Supplement: Supplementary file 4 — Reporting Summary [file 41467_2024_48786_MOESM4_ESM.pdf]

nature portfolio

Corresponding author(s): Xian-Jun Song

Last updated by author(s): May 3, 2024

Reporting Summary

Nature Portfolio wishes to improve the reproducibility of the work that we publish. This form provides structure for consistency and transparency in reporting. For further information on Nature Portfolio policies, see our Editorial Policies and the Editorial Policy Checklist.

Statistics

For all statistical analyses, confirm that the following items are present in the figure legend, table legend, main text, or Methods section.

n/a	Confirmed	
☐	☒	The exact sample size (*n*) for each experimental group/condition, given as a discrete number and unit of measurement
☐	☒	A statement on whether measurements were taken from distinct samples or whether the same sample was measured repeatedly
☐	☒	The statistical test(s) used AND whether they are one- or two-sided<br>*Only common tests should be described solely by name; describe more complex techniques in the Methods section.*
☒	☐	A description of all covariates tested
☒	☐	A description of any assumptions or corrections, such as tests of normality and adjustment for multiple comparisons
☐	☒	A full description of the statistical parameters including central tendency (e.g. means) or other basic estimates (e.g. regression coefficient) AND variation (e.g. standard deviation) or associated estimates of uncertainty (e.g. confidence intervals)
☐	☒	For null hypothesis testing, the test statistic (e.g. *F*, *t*, *r*) with confidence intervals, effect sizes, degrees of freedom and *P* value noted<br>*Give P values as exact values whenever suitable.*
☒	☐	For Bayesian analysis, information on the choice of priors and Markov chain Monte Carlo settings
☒	☐	For hierarchical and complex designs, identification of the appropriate level for tests and full reporting of outcomes
☒	☐	Estimates of effect sizes (e.g. Cohen's *d*, Pearson's *r*), indicating how they were calculated

Our web collection on statistics for biologists contains articles on many of the points above.

Software and code

Policy information about availability of computer code

Data collection	For qPCR data: Bio-Rad CFX Maestro system software;<br>For RNA-seq: Illumina Novaseq;<br>For microscopy: Leica TCS SP5;<br>For LC-MS: Oribtrip Tribrid Lumos mas spectrometer with an EASY-nLC 1200 system (Thermo Fisher);<br>For SEM: Hitachi S-4800;<br>For phenotypes: CanoScan 9000F Mark II;<br>For Western blot: Tanon-5200;<br>For agarose gel image: Tanon-1600.
Data analysis	The gray intensity of protein was analyzed by image J (https://imagej.nih.gov/ij/). The statistical analysis and dada bar graphs were analyzed by microsoft EXCEL 2021 and GraphPad Prism 8.0 version. For RNA-seq data analysis, RNA-seq reads aligned to the rice reference genome (http://rice.uga.edu/index.shtml). Transcription levels of genes were quantified by FPKM (fragments per kilobase of exon per million mapped reads). The criteria of fold-change ≥ 1.5 and q-value ≤ 0.05 were established to identify the differentially expressed genes.

For manuscripts utilizing custom algorithms or software that are central to the research but not yet described in published literature, software must be made available to editors and reviewers. We strongly encourage code deposition in a community repository (e.g. GitHub). See the Nature Portfolio guidelines for submitting code & software for further information.

Data

Policy information about availability of data

All manuscripts must include a data availability statement. This statement should provide the following information, where applicable:

- Accession codes, unique identifiers, or web links for publicly available datasets
- A description of any restrictions on data availability
- For clinical datasets or third party data, please ensure that the statement adheres to our policy

The RNA-seq datasets have been deposited in the NCBI SRA database under accession no. PRJNA979276 (https://dataview.ncbi.nlm.nih.gov/object/PRJNA979276).

Research involving human participants, their data, or biological material

Policy information about studies with human participants or human data. See also policy information about sex, gender (identity/presentation), and sexual orientation and race, ethnicity and racism.

Reporting on sex and gender	N/A
Reporting on race, ethnicity, or other socially relevant groupings	N/A
Population characteristics	N/A
Recruitment	N/A
Ethics oversight	N/A

Note that full information on the approval of the study protocol must also be provided in the manuscript.

Field-specific reporting

Please select the one below that is the best fit for your research. If you are not sure, read the appropriate sections before making your selection.

☒ Life sciences ☐ Behavioural & social sciences ☐ Ecological, evolutionary & environmental sciences

For a reference copy of the document with all sections, see nature.com/documents/nr-reporting-summary-flat.pdf

Life sciences study design

All studies must disclose on these points even when the disclosure is negative.

Sample size	All sample sizes and results of statistical analysis were noted in relevant figure legends or material and methods. Sample sizes were based on previous published studies with similar experiments and were sufficient to show our results were convictive.
Data exclusions	No data exclusion
Replication	All experiments were independently conducted for more than two biological replicates. And the number of biological replications is indicated in the figure legends. All attempts at replication were successful.
Randomization	All plant were grown in the same condition and sample allocation and selection was random.
Blinding	Blinding was not applicable. Data were collected according to the genotype of plants.

Reporting for specific materials, systems and methods

We require information from authors about some types of materials, experimental systems and methods used in many studies. Here, indicate whether each material, system or method listed is relevant to your study. If you are not sure if a list item applies to your research, read the appropriate section before selecting a response.

Materials & experimental systems

n/a	Involved in the study
☐	☒ Antibodies
☒	☐ Eukaryotic cell lines
☒	☐ Palaeontology and archaeology
☒	☐ Animals and other organisms
☒	☐ Clinical data
☒	☐ Dual use research of concern
☐	☒ Plants

Methods

n/a	Involved in the study
☒	☐ ChIP-seq
☒	☐ Flow cytometry
☒	☐ MRI-based neuroimaging

Antibodies

Antibodies used

anti- GST (EASYBIO, Cat#BE2013); anti-Myc (TransGen, Cat#HT101); anti-GFP (TransGen, Cat#HT801); anti-Ub (Santa Cruz, Cat#sc-8017); anti-UbK48 (CST, Cat#8081S); anti-UbK63 (CST, Cat#5621S); anti-MBP (Abclonal, Cat#AE016); anti-His (EASYBIO, Cat#BE2019); anti-Phospho-(Ser/Thr) (Abcam, Cat#ab17464); anti-Actin (EASYBIO, Cat#BE0027); anti-HSP82 (BPI, Cat#AbM51099-31-PU). Secondary antibody: goat-anti-mouse (EASYBIO, Cat#BE0102); goat-anti-rabbit (EASYBIO, Cat#BE0101). The anti-GW6a monoclonal antibody was raised against GW6a.

Validation

The detail information of all antibody can be found in the following list:
anti-GST (http://www.bioeasytech.com/product/2390.html?goods_id=4375);
anti-Myc (https://www.transgen.com/antibody_tag/363.html);
anti-GFP (https://www.transgen.com/antibody_tag/390.html);
anti-Ub (https://www.scbt.com/p/ubiquitin-antibody-p4d1?requestFrom=search);
anti-UbK48 (https://www.cellsignal.cn/product/productDetail.jsp?productId=8081);
anti-UbK63 (https://www.cellsignal.cn/product/productDetail.jsp?productId=5621);
anti-MBP (https://abclonal.com.cn/catalog/AE016);
anti-His (http://www.bioeasytech.com/product/2387.html?goods_id=4372);
anti-Phospho-(Ser/Thr) (https://www.abcam.cn/products/primary-antibodies/phospho-serthr-phe-antibody-ab17464.html);
anti-Actin (http://www.bioeasytech.com/product/2364.html?goods_id=4282);
anti-HSP82 (http://www.proteomics.org.cn/product/202.html);
anti-GW6a was shown to detect GW6a by Gao et al., 2021.

Dual use research of concern

Policy information about dual use research of concern

Hazards

Could the accidental, deliberate or reckless misuse of agents or technologies generated in the work, or the application of information presented in the manuscript, pose a threat to:

No	Yes
☒	☐ Public health
☒	☐ National security
☒	☐ Crops and/or livestock
☒	☐ Ecosystems
☒	☐ Any other significant area

Experiments of concern

Does the work involve any of these experiments of concern:

No	Yes
☒	☐ Demonstrate how to render a vaccine ineffective
☒	☐ Confer resistance to therapeutically useful antibiotics or antiviral agents
☒	☐ Enhance the virulence of a pathogen or render a nonpathogen virulent
☒	☐ Increase transmissibility of a pathogen
☒	☐ Alter the host range of a pathogen
☒	☐ Enable evasion of diagnostic/detection modalities
☒	☐ Enable the weaponization of a biological agent or toxin
☒	☐ Any other potentially harmful combination of experiments and agents

Plants

Seed stocks	Rice varieties: 'Nipponbare' (Oryza sativa L. japonica) and 'Zhonghua 11 (ZH11)' (Oryza sativa L. japonica) were used to generate transgenic recipient as indicated.
Novel plant genotypes	N/A
Authentication	PCR based genotyping and qPCR were used to validate genotypes.
